# Immune and Metabolic Responses in *Ectropis grisescens* Infected by *Metarhizium anisopliae*: Insights from Transcriptome and Metabolome Analyses

**DOI:** 10.3390/insects17030262

**Published:** 2026-02-28

**Authors:** Xiaozhu Wu, Xiaomin Xiong, Muxiang Dai, Juanjuan Cai, Suqing Zhu, Lisi He, Gongmin Cheng, Maosheng Gu, Hao Meng, Feng Wen, Liande Wang

**Affiliations:** 1 College of Resources & Environment, Jiujiang University, Jiujiang 332000, China; wuxiaozhu3@gmail.com (X.W.);; 2 Anhui Chuju Planting and Deep Processing Engineering Research Center, School of Biological Science and Food Engineering, Chuzhou University, Chuzhou 239000, China; 3 State Key Laboratory of Ecological Pest Control for Fujian and Taiwan Crops, Key Laboratory of Biopesticides and Chemical Biology, Ministry of Education, College of Plant Protection, Fujian Agriculture and Forestry University, Fuzhou 350002, China

**Keywords:** tea geometrid, biopesticides, transcriptome and metabolome, immune reactions, amino acid

## Abstract

The tea geometrid (*Ectropis grisescens*) is a significant pest in Chinese tea plantations. Although *Metarhizium anisopliae* serves as an environmentally friendly biocontrol agent against *E. grisescens*, the molecular mechanisms underlying the insect’s immune response remain unclear. This study investigates changes in immunity-related genes and metabolites in *E. grisescens* larvae infected with a virulent strain of *M. anisopliae* through transcriptome sequencing and metabolome analysis. We identified 2409 differentially expressed genes (DEGs) at 48 h post-infection, with 1611 genes up-regulated. GO analysis revealed that 119 DEGs were significantly enriched in immune-related processes. Additionally, 1860 differentially accumulated metabolites (DAMs) were detected, including 652 up-regulated and 1208 down-regulated metabolites, with 236 significantly enriched in 82 KEGG pathways. These findings indicate the activation of immunity-related and detoxifying enzyme-related genes, providing new insights into the physiological and biochemical responses of insects to biopesticides and potential targets for controlling tea geometrid.

## Simple Summary

The tea geometrid (*Ectropis grisescens*) is one of the most destructive defoliators in Chinese tea plantations, resulting in significant economic losses. The overuse of insecticides might lead to a series of food safety problems and environmental issues. Biocides, such as *Metarhizium anisopliae*, are one of the most environmentally friendly alternatives, which have been widely used in agricultural pest control. We isolated and identified a highly virulent strain of *M. anisopliae* against tea geometrid, while its insecticidal molecular mechanism remains unclear. Here, we combined transcriptome and metabolome analysis, analyzing the changes of immunity-related genes and metabolites in *E. grisescens* that were infected by *M. anisopliae*, which provide molecular insights into the insect–pathogen interaction.

## 1. Introduction

The tea plant (*Camellia sinensis*) is a crucial economic crop and one of the world’s most popular non-alcoholic beverages [1,2]. Among the insect pests causing damage to tea plants, the tea geometrid, tea green leafhoppers, tea mosquito bugs, and Paraguay tea ampul pose significant threats to tea plantations [3,4,5,6,7,8]. *Ectropis grisescens*, commonly known as tea geometrid, with an arched shape, which is a notorious pest in tea plantations of East Asia, causes substantial economic losses by feeding on tender leaves. *E. grisescens* belongs to the Geometridae family, a group of moths characterized by their distinctive looping caterpillar motion, hence their alternative name tea looper [5,9]. The species, which is native to China, has garnered attention for its impact on tea cultivation, where it poses a considerable economic threat by feeding on tender leaves [3].

For decades, spraying chemical and biochemical insecticides has been the most efficient way to control outbreaks of tea geometrids. However, frequent use of these insecticides has led to increased tolerance or resistance in many insects, and associated risks of health, safety, and environmental contamination [10,11]. Therefore, to meet the safety challenge of chemical and biochemical pesticides, an efficient, economical, and environmentally friendly pest control method was required. In this regard, biological control using entomopathogenic fungi (EPFs) has emerged as an effective strategy [12,13,14], as these fungi exert their pest-control effects by infecting hosts and triggering specific pathological responses. Specifically, in lepidopteran larvae, infection by EPFs can induce a typical sharp mycosis, which is characterized by rapid fungal proliferation in the hemocoel and high host mortality within a short period [15]. Following intrahemocoelic invasion, fungal propagules quickly colonize the host, triggering a rapid and intense immune response that determines the outcome of infection [16].

To date, more than 40 entomopathogenic fungi effective against insect and mite pests of tea have been documented, which belong to four phyla: Zygomycotina, Ascomycotina, Basidiomycotina, and Deuteromycotina [17]. Among these EPFs, *Metarhizium anisopliae* was specifically selected for this study owing to its strong pathogenicity to tea geometrids, remarkable control efficacy in laboratory and preliminary field assays, and great potential as a commercial biocontrol agent for tea pest management. Notably, *M. anisopliae* disseminates in the environment and colonizes hosts primarily via conidia. These conidia attach to the host’s cuticle, germinate and penetrate into the hemocoel to initiate infection [18]. Given that tea is one of the most widely consumed beverages globally, understanding the host−pathogen interactions between tea geometrids and EPFs (e.g., *M. anisopliae*) is crucial for advancing sustainable agriculture and pest management practices.

EPFs infected their hosts through the exoskeleton or cuticula and were pathogenic to both soft- and hard-bodied insects, as well as a range of other arthropods, including ticks and mites. This mode of infection contrasts with those of insect-pathogenic bacteria and viruses, which typically infect through other mechanisms [12,19]. For example, the spores of *M. anisopliae* began the infection processes on cuticles, including spore germination, host recognition, and the formation of penetrating structures [20,21]. This infection process was a typical host−pathogen interaction (HPI). In the long-term evolution, insects have developed robust immune defenses against fungal infections [22]. Generally, insects respond to fungal pathogen invasion by triggering two types of innate immune reactions: the cellular and the humoral responses, which involve large amount of gene expression, protein, and metabolite synthesis [23,24,25]. For instance, cellular reactions include phagocytosis, hemocyte aggregation, and pathogen encapsulation, while humoral reactions include the production of antimicrobial peptides (AMPs), complex enzyme cascades that regulate the activation of immune-related molecular precursors, such as prophenoloxidase (PPO), Spaetzle/Toll, and proplasmatocyte-spreading peptide (proPSP), production of humoral pattern recognition receptors (PRRs) and lyases, as well as reactive oxygen/nitrogen intermediates (ROIs or RNIs) [24,26,27,28,29,30]. Therefore, studying these interactions provides valuable insights into the co-evolution of EPFs and their insect hosts, improving our understanding of EPF aggressiveness and toxicity towards insects.

Compared to traditional research methods, omics approaches, like large-scale comparative transcriptomics, proteomics, and metabolomics, have the potential to shed unprecedented insights into the global gene networks and molecular regulations of metabolism involved in biological processes [31,32,33]. Over the years, many insect transcriptomes have been sequenced to examine the expression levels of mRNA transcripts in various organisms, organs, and tissues [34,35,36]. For example, Xiong et al. performed an immune transcriptome analysis of cotton bollworm *Helicoverpa armigera* larval hemocytes and fat body in response to the challenges of entomopathogenic fungi *B. bassiana* and Gram-negative bacteria *Enterobactor cloacae*, and identified 233 significantly differentially expressed immune-related genes, including the putative IMD and JAK-STAT pathway members, which were divided into pattern recognition, signal transduction, execution, and cellular responses [37]. The transcriptome of *Spodoptera exigua* larvae exposed to different types of microbes identified 186 unigenes with homology to immune-related genes [38]. An RNA-seq approach showed that a group of genes encoding cuticular proteins, serine protease inhibitors, AMPs, and toxin detoxification may be associated with the resistance of silkworm-resistant strains in response to *Beauveria bassiana* infection [39]. The transcriptome analysis showed that *Hepialus xiaojinensis* larvae in the 3rd instars could resist the invasion of *Ophiocordyceps* species by immune and nervous systems [40]. On the other hand, due to its holistic characteristics, metabolomic analysis provided a powerful new tool for studies of the adaptive responses of insects, such as environmental changes, pathophysiological stimuli, genetic modifications, and host−pathogen interactions [41,42,43,44]. Using LC-MS- and GC-MS-based metabolomic approaches, a total of 115 differential metabolites, including fatty acids, amino acids, phospholipids, bioamines, and other compounds, were identified in honey bees after exposure to thiacloprid [45]. Additionally, metabolomics could also provide insights into the responses of primary and secondary metabolic pathways in host insects during pathogenic microorganism infection [46,47,48]. For example, 93 differential metabolites were identified in the *B. bassiana*-infected *Spodoptera frugiperda* larvae, revealing that *B. bassiana* infection might affect purine metabolism, arginine biosynthesis, butanoate metabolism, and phenylalanine metabolism of *S. frugiperda* larvae [49]. A metabolomic analysis of dead caterpillars showed that a fungal consortium might suppress the caterpillars’ immune system and insecticide action due to its antioxidant mechanism [50]. In general, transcriptomics and metabolomics provided plausible explanations for the biological functions of insects caused by insecticides and pathogenic microorganism stress.

In recent years, genome-wide transcriptome and metabolome analyses have been used to investigate the molecular mechanisms and biological processes involved in the development of insect pests and their responses to insecticides [51,52,53,54]. However, little was known about the changes in insect pest transcriptomes and metabolomes in response to fungal biopesticide [49]. Previously, we characterized and examined the expression profiles of MAPK cascade genes in *E. grisescens* in response to *M. anisopliae*, suggesting a conserved MAPK architecture for the immune signal transduction pathway [5]. To observe how metabolome and its underlying transcriptomic regulation were triggered during *E. grisescens* in response to *M. anisopliae*, the changes of immunity-related gene and metabolic material in *E. grisescens* larvae infected by a highly virulent strain of *M. anisopliae* were investigated using transcriptome sequencing and metabolome analysis. Our analysis revealed a complicated response system of tea geometrid in response to *M. anisopliae*, including activation of immune-related processes, metabolism of xenobiotics by cytochrome P450, and amino acid synthesis. Understanding these physiological and biochemical reactions can provide potential targets for controlling tea geometrid pests using biopesticides.

## 2. Materials and Methods

### 2.1. Insect Rearing and Infection Bioassays

*E. grisescens* moths were obtained from a laboratory colony at the Tea Research Institute, Chinese Academy of Agricultural Sciences, Hangzhou, China. The larvae were reared on fresh tea leaves under controlled conditions (23 ± 2 °C, 70–80% relative humidity, 16 h light/8 h dark photoperiod) in an insect-rearing laboratory. The *M. anisopliae* strain used in this study was a highly pathogenic strain against tea geometrids, isolated from naturally infected tea pests in tea plantations of Fuzhou and preserved in our laboratory; it was routinely activated and cultured on potato dextrose agar (PDA) medium at 28 °C for 10 d to obtain conidia for suspension preparation. Fourth-instar larvae were randomly selected for microinjection, with each larva receiving a 2 µL injection of either *M. anisopliae* conidial suspension (5 × 10^7^ conidia mL^−1^) or 0.05% sterile Tween-80 solution (as the control) via a microliter syringe (Shanghai GaoGe Co., Ltd., Shanghai, China). Injections were administered into the hemocoel through the intersegmental membrane between the 3rd and 4th abdominal segments, a standard inoculation site for lepidopteran larvae that facilitates rapid colonization and infection initiation of fungal conidia in the host body. The concentration of the *M. anisopliae* conidial suspension was set according to our previous pathogenicity pre-experiments, corresponding to the median lethal concentration (LC_50_) of this fungal strain against fourth-instar tea geometrid larvae. A concentration gradient infection assay was conducted to determine the LC_50_ of *M. anisopliae* conidia against *E. grisescens* larvae. Briefly, a series of gradient concentrations of *M. anisopliae* conidial suspension were prepared, and healthy *E. grisescens* larvae with uniform growth status were selected for intrahemocoel injection infection, with a blank control group setup (injected with sterile Tween-80 solution). Each treatment group had three biological replicates with 100 larvae per replicate. The number of dead larvae in each group was recorded at 48 h post-infection (hpi), and the mortality rate was calculated. The LC_50_ and its 95% confidence interval were statistically calculated using the probit analysis module in IBM SPSS Statistics v21.0 software (Tables S1 and S2). The numbers of dead larvae were recorded for 10 days; during this period, dead larvae were collected and incubated under moist conditions to observe hyphal emergence from the cadavers. At 24 h and 48 hpi, larvae from each treatment and the control group were collected for transcriptome and metabolome sample preparation, with larval whole bodies used after careful removal of intestinal contents. At each sampling time point, each experimental group comprised 9 biological replicates (30 larvae per replicate); 3 replicates were designated for transcriptome analysis, and the remaining 6 were for untargeted metabolomics. All samples were stored at −80 °C after liquid nitrogen freezing if not immediately used for RNA isolation and subsequent analysis. Transcriptome and metabolome analysis were performed by Biomarker Technologies Co., Ltd. (Beijing, China).

### 2.2. RNA Extraction and Illumina Sequencing

The total RNA was extracted from each sample with the TRIzol method. The purity, concentration, and integrity of RNA samples were examined by NanoDrop (Thermo Fisher Scientific, Wilmington, DE, USA), Qubit 2.0 (Thermo Fisher Scientific, Wilmington, DE, USA), and Agilent 2100 (Agilent Technologies, Santa Clara, CA, USA), respectively. The qualified cDNA library was uploaded on the Illumina NovaSeq 6000 platform (Illumina, San Diego, CA, USA) for sequencing with a read length of PE150. Raw reads were inferred from light intensity signals generated by the Illumina sequencing platform through base calling analysis, which were stored in FASTQ (fq) file format.

### 2.3. Assembly and Annotation of Transcripts

Low-quality sequences (quality < 20) and adapter sequences were removed. The clean reads were aligned to the reference genome *E. grisescens* (Access No. PRJNA660825) using HISAT2 software (v2.2.1) [55]. The mapping files were compressed and sorted by using Samtools (v1.19), and then, read assembly and abundance estimation were performed by using StringTie software (v2.2.3) [56]. In this experiment, the fragments per kilobase of exon model per million mapped fragments (FPKMs) was used to indicate the relative expression of the gene, and an FDR of <0.05 was used to determine whether it was a differentially expressed gene (DEG). The DESeq2 R package (version 1.42.1) was performed to screen DEGs, and the screening criteria were FDR < 0.05 and |log_2_FoldChange| ≥ 1. GO (gene ontology) and KEGG (Kyoto Encyclopedia of Genes and Genomes) pathway enrichment were performed using the clusterProfiler R package (v4.8.1), and pathways irrelevant to insect biological processes were further screened out from the KEGG enrichment results to ensure the biological rationality of the subsequent functional interpretation of DEGs.

### 2.4. Metabolite Extraction

An amount of 50 mg insect sample was transferred to an Eppendorf tube, and 1000 μL of extraction solution containing the internal standard [methanol/acetonitrile/water (2:2:1, *v*/*v*), internal standard (2-Chloro-L-phenylalanine) concentration 20 mg/L] was added, swirled and mixed well for 30 s. After homogenization, the mixture was shaken at 45 Hz for 10 min, allowed to stand at −20 °C for 1 h and then centrifuged the mixture at 13,400× *g* at 4 °C for 10 min, and 500 µL of the supernatant was transferred into another centrifuge tube. After vacuum drying, 160 μL extraction solution (acetonitrile/water, 1:1, *v*/*v*) was added to the dried metabolites for re-dissolution. The supernatant was taken into the sample bottle for LC-MS/MS analysis after centrifugation (13,400× *g*).

### 2.5. LC-MS/MS Analysis

The LC/MS system for metabolomics analysis is composed of an ultra-high-performance liquid chromatography system (Waters UPLC Acquity I-Class PLUS; Waters Corporation, Milford, MA, USA) and a high-resolution mass spectrometer (Waters UPLC Xevo G2-XS QT; Waters Corporation, Milford, MA, USA). Acquity UPLC HSS T3 column (1.8 μm 2.1 × 100 mm; Waters Corporation, Milford, MA, USA) was used to separate the derivatives in electrospray ionization (ESI) positive (POS) and negative (NEG) ion modes. In each data acquisition cycle, dual-channel data acquisition was carried out for both low and high collision energies. The low collision energy was 2 V, the high collision energy range was 10−40 V, and the scanning frequency was 0.2 s for a mass spectrum. The parameters of the ESI ion source were as follows: capillary voltage: 2000 V (POS) or −1500 V (NEG); cone voltage: 30 V; ion source temperature: 150 °C; de-solvent gas temperature: 500 °C; backflush gas flow rate: 50 L/h; de-solvent gas flow rate: 800 L/h [57].

### 2.6. Statistical Analysis of Metabolomics Data

The raw data collected by MassLynx V4.2 were processed by Progenesis QI software (v2.4) for peak extraction, alignment, and other data processing operations. The identification was carried out based on the Progenesis QI software online METLIN and Biomark’s self-built library, and at the same time, theoretical fragment identification was also carried out. The mass number deviation of the parent ion was 100 ppm, and the mass number deviation of the fragment ion was less than 50 ppm. The identified compounds were searched for classification and pathway enrichment in KEGG, HMDB, and lipid maps databases. Finally, statistical analysis was carried out by the R program, including principal component analysis (PCA), partial least squares discriminant analysis (PLS-DA), and orthogonal partial least squares discriminant analysis (OPLS-DA). The different metabolites of KEGG pathway enrichment significance were calculated using a hypergeometric distribution test.

### 2.7. Transcriptome and Metabolome Association Analysis

Transcriptome and metabolome association analyses were conducted using a custom Python 3.8+ script to identify shared KEGG pathways between DEGs and DAMs. Two KEGG-annotated input datasets were prepared for the analysis: the DEG dataset included gene IDs, corresponding KEGG Orthology (KO) terms, and mapped KEGG pathway IDs and names, while the DAM dataset contained metabolite IDs, KEGG Compound IDs, and matched KEGG pathway information. With the Pandas library (v1.3.5), we preprocessed the two annotation tables, extracted non-redundant KEGG pathway ID lists from the DEG and DAM datasets separately, and calculated the intersection of the two lists to screen for common pathways enriched in both transcriptomic and metabolomic responses to *M. anisopliae* infection; the resulting shared pathways were then annotated with full functional information and exported for downstream functional interpretation.

## 3. Results

### 3.1. Virulence of M. anisopliae Against E. grisescens

**Figure 1:**
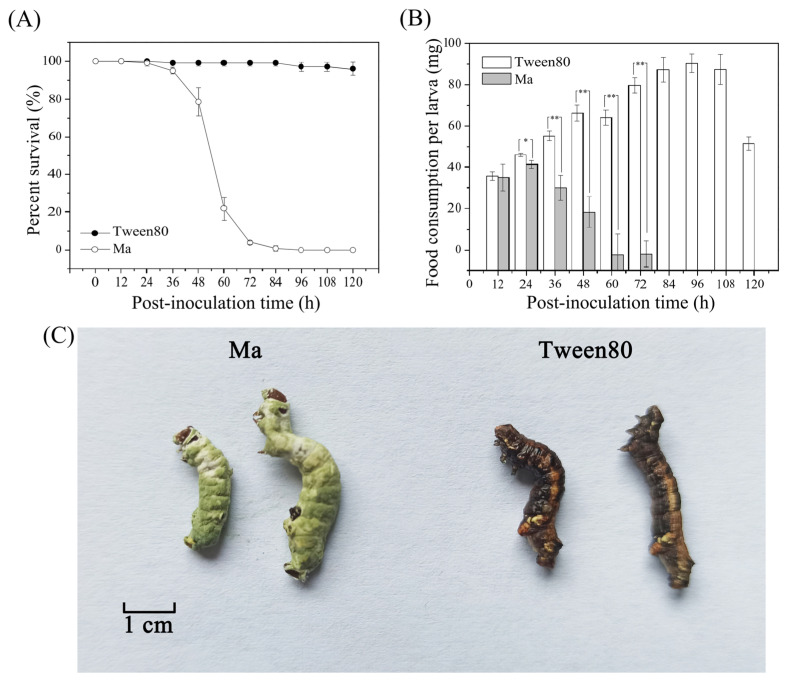
(**A**) Effects of *M. anisopliae* conidial suspension inoculation on the survival rate of *E. grisescens* larvae. Survival rate data are presented for the first 5 days post-injection as no significant changes in larval survival rate were observed thereafter. (**B**) Effects of *M. anisopliae* conidial suspension inoculation on the feeding capacity of *E. grisescens* larvae (*, *p* < 0.05; **, *p* < 0.01). (**C**) Different phenotypes of *E. grisescens* larvae after inoculation with *M. anisopliae* conidial suspension and 0.05% sterile Tween-80 (control). The control larvae in (**C**) showed body surface color change due to approaching the prepupal stage, which is a normal physiological phenomenon.

To assess the virulence effect of *M. anisopliae* against *E. grisescens*, the fourth-instar *E. grisescens* larvae were randomly selected to be inoculated with *M. anisopliae*, and the feeding capacity and survival rate of *E. grisescens* within 5 d after inoculation were calculated. The *E. grisescens* larvae began to die 48 h after *M. anisopliae* inoculation. According to statistics, over 78% of *E. grisescens* larvae were killed by the *M. anisopliae* infection after 60 h, while almost all *E. grisescens* larvae were killed after 84 h; eventually, 100% mortality was reached by 96 hpi, showing typical symptoms of sharp mycosis (Figure 1A). From 48 hpi onwards, the number of *E. grisescens* larvae killed by *M. anisopliae* was significantly higher than that in the Tween-80 group after 48 hpi (*p* < 0.01). In contrast, over 90% of the control larvae successfully eclosed into adults by the end of the experiment, confirming their normal development. The feeding capacity of *E. grisescens* larvae also decreased significantly at 36 hpi (*p* < 0.01), with almost no feeding at 60 hpi (Figure 1B). After inoculation of *M. anisopliae* spores, the larvae died and became stiff, and the white hyphae on the surface were visualized at 5 d post-inoculation. Three days later, hyphae covered the entire larvae and the cadavers turned green (Figure 1C).

### 3.2. Transcriptome Analysis of E. grisescens in Responses to M. anisopliae

**Figure 2:**
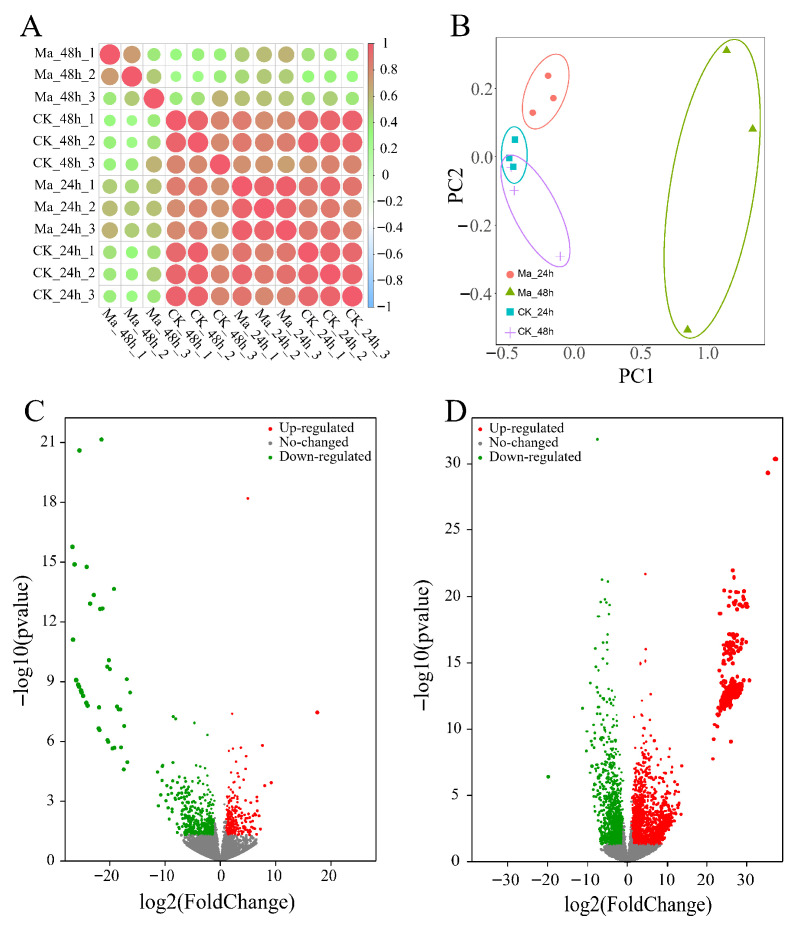
Tanscriptome response of *E. grisescens* in responses to *M. anisopliae*. (**A**) Pearson’s correlation coefficient analysis. The color of each dot represents the Pearson’s correlation coefficient, ranging from −1 (blue, negative correlation) to 1 (red, positive correlation). The size of each dot is proportional to the absolute value of the correlation coefficient, with larger dots indicating a stronger correlation. (**B**) Principal component analysis (PCA). (**C**,**D**) Volcano maps of differentially expressed genes in *E. grisescens* larvae 24 h (**C**) and 48 h (**D**) after *M. anisopliae* infection. The significant DEGs with |log_2_FoldChange| ≥ 1 and FDR < 0.05 are represented by green (down-regulated) and red (up-regulated) dots.

To examine the transcriptome profile of *E. grisescens* larvae in response to *M. anisopliae*, four samples [Tween80 treatment after 24 h (CK_24h) and 48 h (CK_48h), *M. anisopliae* treatment after 24 h (Ma_24h) and 48 h (Ma_48h)] in three replicates were used to construct 12 cDNA libraries. As a result, a total of 284.50 M reads (85.20 Gb) of clean data were obtained with a minimum yield of 5.91 Gb clean data per sample; in turn, the clean reads were aligned with an annotated reference genome (assembly GCA_017562165.1, Genbank Accession no. PRJNA660825) (Table 1). The evaluation of the correlation of biological replicates in the control and treatment groups was determined by Pearson’s correlation coefficient analysis (Figure 2A). The principal component analysis (PCA) results showed the *M. anisopliae* treatment groups were well separated, suggesting significant differences existing in gene expression between the treatment and control groups (Figure 2B). Volcano plots were constructed based on *p*-values and fold changes to quantify the significantly differentially expressed genes (DEGs) in *E. grisescens* following *M. anisopliae* infection. Based on the threshold of |log_2_FoldChange| ≥ 1 and a *p*-value of <0.05, 798 and 76 DEGs were up-regulated, and 1611 and 21 DEGs were down-regulated in the Ma_24h and Ma_48h groups, respectively (Figure 2C,D).

**Figure 3:**
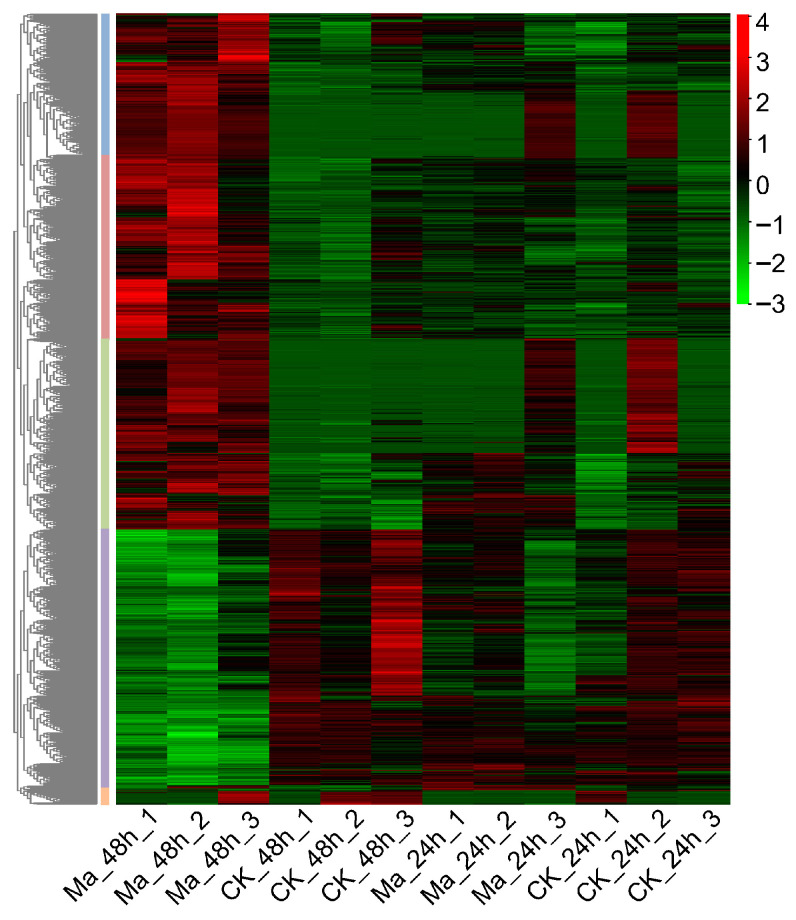
Cluster analysis heatmap of DEGs in *E. grisescens* larvae at 24 hpi and 48 hpi following *M. anisopliae* infection.

To compare gene profiles of the *M. anisopliae*-infected larvae in different treatment periods, a hierarchical clustering analysis of the DEGs was performed using fragments per kilobases per million mapped reads by TBtools (v2.156) [58]. Five major gene clusters were identified that exhibited distinct expression patterns among the different groups (Figure 3). It was revealed that three major gene clusters showed a relatively high expression level in the Ma_48h group, while the expression profiles were not significantly different among Ma_24h, CK_24h, and CK_48h groups.

**Figure 4:**
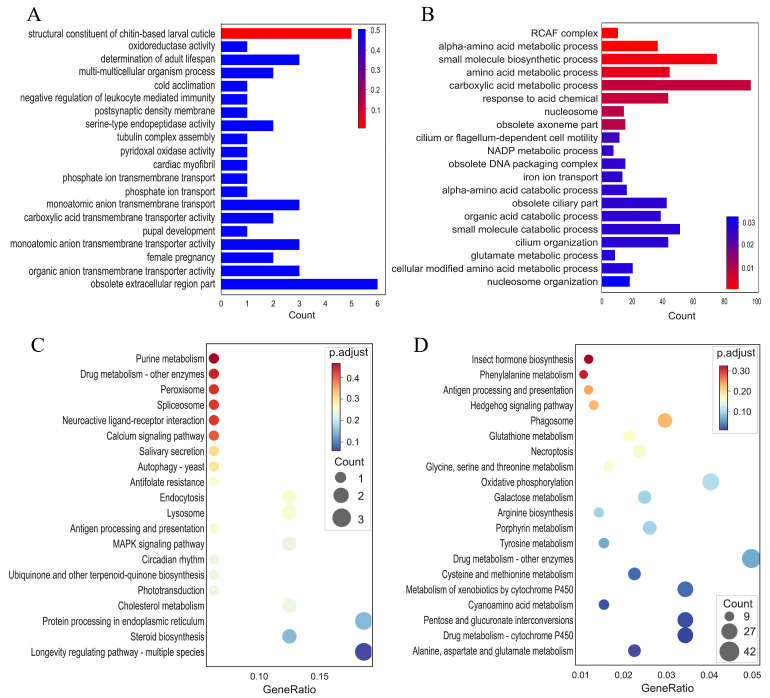
Gene ontology (GO) annotation ((**A**) 24 hpi; (**B**) 48 hpi) and Kyoto Encyclopedia of Genes and Genomes (KEGG) enrichment ((**C**) 24 hpi; (**D**) 48 hpi) of DEGs in *E. grisescens* larvae at 24 hpi and 48 hpi following *M. anisopliae* infection. Colors correspond to the adjusted *p*-value (*p*.adjust), with a gradient from red to blue. The color bar showing the scale of *p*.adjust is presented on the right.

To further explore the biological function of the DEGs between the control and treatment groups, GO analysis was performed to distribute the DEGs into three major functional categories: Cell Component (CC), Molecular Function (MF), and Biological Process (BP). The top 20 GO terms of the DEGs between the control and EPF treatment groups are shown in Figure 4A,B. The results of the top 20 GO terms showed that the DEGs of CK_24h_vs_Ma_24h were significantly enriched in the obsolete extracellular region part (GO:0044421) in the cellular component (CC) category, whereas the structural constituent of the chitin-based larval cuticle (GO:0008010) was the most enriched GO term in the molecular function (MF) category. The DEGs of CK_48h_vs_Ma_48h were significantly enriched in the carboxylic acid metabolic process (GO:0019752) and small molecule biosynthetic process (GO:0044283) in the biological process (BP) category, whereas the obsolete ciliary part (GO:0044441) was the most enriched GO term in the CC category. The KEGG enrichment analysis of DEGs was further determined by clusterProfiler to investigate the response of different processes in the control and EPF treatment groups. Further, the sets of DEGs were assigned to significant KEGG pathways (*p* > 0.05), while the top 20 categories enriched in the KEGG pathways were presented (Figure 4C,D). As shown in the results, the longevity-regulating pathway (ko04213), steroid biosynthesis (ko00100), protein processing in endoplasmic reticulum (ko04141), cholesterol metabolism (ko04979), and MAPK signaling pathway (ko04010) were the top five significantly enriched pathways for *M. anisopliae* treatment after 24 h, and the other pathways were primarily related to lysosome (ko04142) and endocytosis (ko04144). For the *M. anisopliae* treatment after 48 h, the top five most enriched pathways were drug metabolism (ko00983), oxidative phosphorylation (ko00190), drug metabolism–cytochrome P450 (ko00982), pentose and glucuronate interconversions (ko00040), and metabolism of xenobiotics by cytochrome P450 (ko00980). Moreover, we observed a range of important immunity-related genes and detoxification enzyme genes after *M. anisopliae* infection, for example, gram-negative binding proteins (*GNBP*), β-1,3-glucan recognition protein (*βGRP*), peptidoglycan recognition protein (*PGRP*), serine protease (*SP*), *serpins*, *Spätzle* (*Spz*), *AMPs*, *PPO*, cytochrome P450 (*CYP450*), etc. (Table 2). Specifically, we identified four AMP genes in *E. grisescens*: 1 *attacin* gene, 1 *gloverin* gene, and 2 *cecropin* genes. Among these, the *attacin* and *gloverin* genes were significantly up-regulated (*p* < 0.05 or *p* < 0.01) consistently at both 24 and 48 hpi following *M. anisopliae* infection, whereas the two *cecropin* genes were significantly up-regulated (*p* < 0.01) exclusively at 48 hpi (Figure S1). Notably, other AMP genes previously documented in moths did not exhibit sustained significant differential expression within the 24–48 hpi time window analyzed in this study.

### 3.3. Metabolomics Profiles of E. grisescens in Responses to M. anisopliae

**Figure 5:**
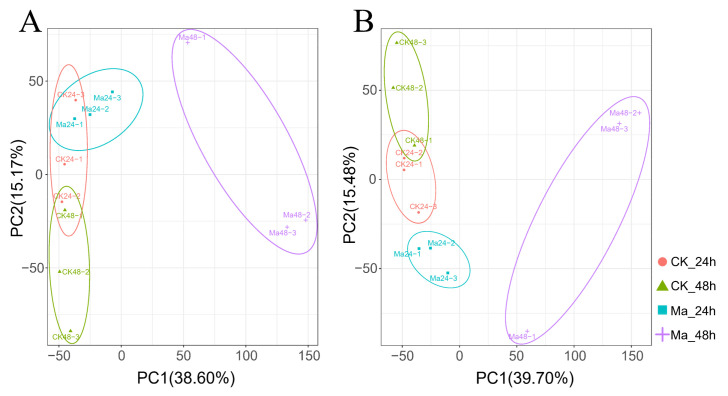
Metabolic profile of *E. grisescens* in responses to *M. anisopliae*. Principal component analysis (PCA) plots of metabolism identified by LC-MS/MS of CK_24h, CK_48h, Ma_24h, and Ma_48h in POS ion mode (**A**) and NEG ion mode (**B**).

Untargeted metabolomics analyses in both electrospray ionization (ESI) positive (POS) and negative (NEG) ion modes were performed on *E. grisescens* larvae in response to *M. anisopliae* using an LC-MS/MS platform. The principal component analysis (PCA) was performed to analyze the metabolic profiles and to understand the overall difference between the treatment and control groups, as well as the variability within groups (Figure 5A,B). The PCA results showed a no-complete separation between CK_24h and CK_48h groups in both POS and NEG ion modes. The Ma_24h group showed a complete separation from other groups in NEG ion mode, while no complete separation was observed in POS ion mode. The PCA models obtained from the Ma_48h group showed a complete separation with other groups in both POS and NEG ion modes, indicating a significant difference in metabolic characteristics.

**Figure 6:**
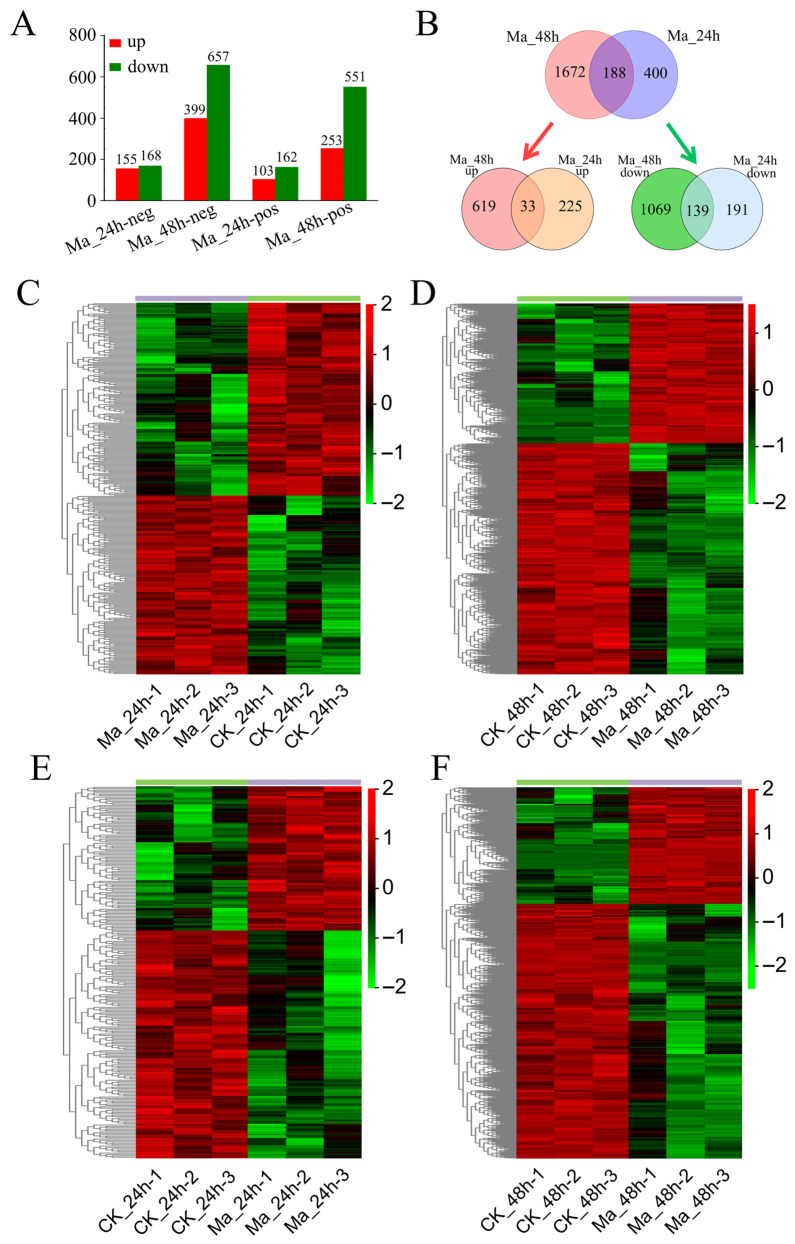
Analysis of DAMs in *E. grisescens* in responses to *M. anisopliae*. (**A**) Numbers of metabolites that were up-regulated (red) and down-regulated (green) in *M. anisopliae*-infected *E. grisescens* larvae. (**B**) Venn diagram illustrating the overlap of DAMs at the two time points. (**C**–**F**) Heatmaps of DAMs in *E. grisescens* larvae in negative ion mode (**C**,**D**) and positive ion mode (**E**,**F**) after *M. anisopliae* infection. The red color represents the relative level of the up-regulated metabolites, and the green color represents the down-regulated metabolites.

As statistical results based on metabolomics analyses in both POS and NEG ion modes, combining the results of *p*-values of < 0.05 and |log_2_FoldChange| ≥ 1 as thresholds for significant differences, a total of 588 metabolites in *E. grisescens* larvae were significantly changed after *M. anisopliae* infection 24 h, while 1860 metabolites were significantly changed after 48 h (Figure 6A). Among these, a total of 103 metabolites were significantly up-regulated, and 162 metabolites were significantly down-regulated in POS ion mode in *E. grisescens* larvae 24 h after *M. anisopliae* infection, while 155 metabolites and 168 metabolites were significantly up- and down-regulated in NEG ion mode in the Ma_24h group, respectively. In *E. grisescens* larvae 48 h after *M. anisopliae* infection, a total of 253 and 399 metabolites were significantly up-regulated in POS and NEG ion modes, respectively, while 551 and 657 metabolites were significantly down-regulated in POS and NEG ion modes, respectively (Figure 6B). In addition, we have further identified metabolites that are associated with *M. anisopliae* treatment (Figure 6C–F). After 48 h of *M. anisopliae* infection, most metabolites were significantly down-regulated compared to the control samples in both NEG and POS ion modes (Figure 6D,F); however, less differentially accumulated metabolites (DAMs) were identified after 24h of *M. anisopliae* infection (Figure 6C,E). Besides, among 588 DAMs identified in CK_24h_vs_Ma_24h, 258 were up-regulated, and 330 were down-regulated, including 4 alkaloids, 34 benzenoids, 1 hydrocarbon, 2 lignans, 135 lipids, 22 nucleosides and nucleotides, 92 organic acids and derivatives, 8 organic nitrogen compounds, 41 organic oxygen compounds, 79 organo heterocyclic compounds, 57 phenylpropanoids and polyketides, and 113 other metabolites. In CK_48h_vs_Ma_48h, the following DAMs (652 up- and 1208 down-regulated) were identified: 14 alkaloids, 101 benzenoids, 3 hydrocarbons, 6 lignans, 402 lipids, 72 nucleosides and nucleotides, 282 organic acids and derivatives, 19 organic nitrogen compounds,166 organic oxygen compounds, 250 organo heterocyclic compounds, 4 organosulfur compounds, 165 phenylpropanoids and polyketides, and 376 other metabolites.

**Figure 7:**
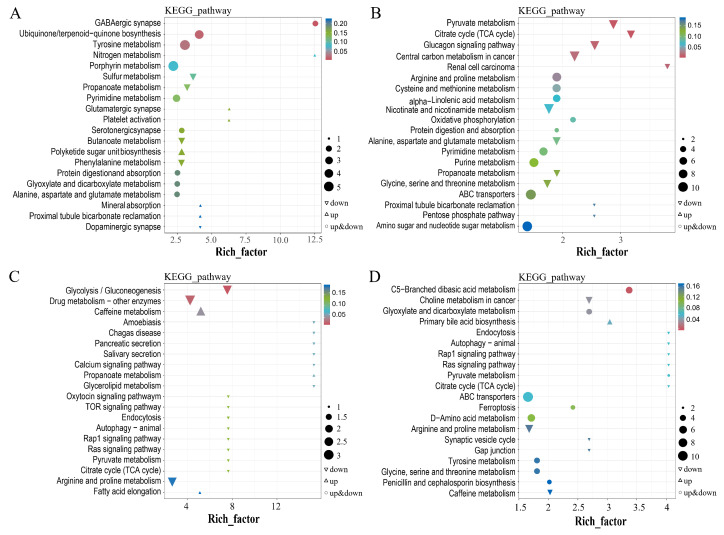
KEGG functional analysis of DAMs in *E. grisescens*, in negative ion mode (**A**,**B**) and positive ion mode (**C**,**D**) after *M. anisopliae* infection for 24 h (**A**,**C**) and 48 h (**B**,**D**).

To obtain more information on the physiological changes of *E. grisescens* larvae in response to *M. anisopliae*, a KEGG enrichment analysis based on the results of DAMs was performed to explore the functions of the metabolites that were altered in *E. grisescens* larvae after *M. anisopliae* infection. The most significantly modified pathways between the Ma_24h and CK_24h groups were the metabolism of amino acids, nitrogen metabolism (ko00910), ubiquinone and other terpenoid-quinone biosynthesis (ko00130), glycolysis/gluconeogenesis (ko00010), and drug metabolism (ko00983) (Figure 7A,C). The changes in pyruvate metabolism (ko00620), glucagon signaling pathway (ko04922), C5-branched dibasic acid metabolism (ko00660), choline metabolism (ko05231), and endocytosis (ko04144) were characterized in the Ma_48h group. The results indicated that most DAMs in *E. grisescens* larvae in responses to *M. anisopliae* at 24 h and 48 h were both significantly enriched in many pathways, such as alanine, aspartate, and glutamate metabolism (ko00250), autophagy-animal (ko04140), caffeine metabolism (ko00232), citrate cycle (ko00020), glyoxylate and dicarboxylate metabolism (ko00630), propanoate metabolism (ko00640), protein digestion and absorption (ko04974), rap1 signaling pathway (ko04015), Ras signaling pathway (ko04014), tyrosine metabolism (ko00350) (Figure 7B,D). Moreover, the significant metabolic pathways, including the AMPK signaling pathway (ko04152), amino acid metabolism, reactive oxygen species (ko05208), and metabolism of xenobiotics by cytochrome P450 (ko00980), were summarized to reveal the effects of the *M. anisopliae* in *E. grisescens* larvae (Table 3).

### 3.4. Transcriptome−Metabolome Association Analysis of Common KEGG Pathways

To systematically elucidate the synergistic regulatory crosstalk between genes and metabolites during the response of *E*. *grisescens* to *M*. *anisopliae* infection, we performed KEGG pathway enrichment association analysis on DEGs and DAMs. A total of 22 common significantly enriched KEGG pathways were identified in the transcriptome and metabolome datasets (Figure S2). Among these pathways, cytochrome P450-related functional modules exhibited the highest enrichment significance and the most abundant molecular participation in the association analysis: drug metabolism—cytochrome P450 (ko00982) and metabolism of xenobiotics by cytochrome P450 (ko00980). This indicates that members of the CYP450 family were significantly up-regulated at the transcriptional level, and their associated metabolites underwent coordinated expression/accumulation changes. Collectively, these findings confirm that CYP450-mediated detoxification metabolic pathways represent the core regulatory nodes underlying the immune/detoxification response of *E. grisescens* to *M. anisopliae* infection. In addition, multiple amino acid metabolism-related pathways were identified as common significantly enriched pathways, including tyrosine metabolism (ko00350), alanine, aspartate and glutamate metabolism (ko00250), cysteine and methionine metabolism (ko00270), and glutathione metabolism (ko00480). These pathways mediate the synthesis and catabolism of amino acids in insects, while also providing critical substrates for immune processes such as AMP synthesis and antioxidant defense. This highlights the pivotal role of metabolic reprogramming in orchestrating the antifungal immune response of *E. grisescens*.

## 4. Discussion

In recent years, combined transcriptomic and metabolomic approaches have become core tools for deciphering the molecular mechanisms of insect growth, development, and stress responses, particularly the interactions between insect hosts and entomopathogenic fungi [52,59,60]. Tea geometrid is known as a notorious pest of tea plants, causing serious economic losses; however, the molecular mechanisms underlying its response to fungal biopesticides remain poorly understood. To address this, we treated *E. grisescens* larvae via intrahemocoel injection of *M. anisopliae* conidia, to dissect the host’s response to fungal infection. Preliminary experiments confirmed that the conidia used in this approach germinate efficiently and colonize the host hemocoel, leading to sharp mycosis, a typical pathological process induced by entomopathogenic fungi, with 100% larval mortality observed at 96 hpi. Based on the progression of sharp mycosis, we selected 24 hpi and 48 hpi as sampling time points to capture the host’s molecular response during the active progression of fungal infection. Our analyses identified a large number of DEGs and DAMs between the *M. anisopliae*-infected group and controls, which enabled us to characterize the regulatory dynamics of *E. grisescens* in response to fungal challenge.

Insect hosts rely on innate immune defense systems to recognize and respond to invading pathogenic microorganisms [61,62,63]. In general, four signaling pathways were investigated in insects, which mediate immune responses to different pathogens: Jak/STAT (Janus kinase/signal transducer and activator of transcription) signal pathway, Toll signal pathway, Imd (Immunodeficiency) signaling pathway, and JNK (c-Jun NH(2)-terminal protein kinases) signaling pathway [63,64]. On the other hand, significant changes occurred in the activation of detoxifying enzyme-related genes, including glutathione transferase (*GSTs*), *CYP450*, carboxylesterase (*CES*), juvenile hormone-binding protein (*JHBP*), and heat shock protein (*HSP*) [65]. In this study, through transcriptomic analysis, we identified a large number of immunity-related DEGs in *E. grisescens* larvae following infection with *M. anisopliae*. According to KEGG functional analysis and GO annotation of *E. grisescens* transcriptome, the identified immune-related genes of *E. grisescens* were classified, which were involved in recognition, signal regulation, intracellular signal transduction, and effector function, indicating that *E. grisescens* has a perfect immune defense system. Transcriptomic results showed that large change genes were significantly enriched in pattern recognition receptor genes (including *PGRP*, *GRP*, and *GNBP*), *SP*, *serpins*, protein and steroid biosynthesis pathway, and MAPK signaling pathway within 24 h after *M. anisopliae* infection, as well as *CYP450s*, implying that *E. grisescens* have already initiated an immune response, such as intracorporal pattern recognition and melanization reaction, in the early stages of infection [66,67,68]. Notably, we found that *serpins* were significantly up-regulated at both 24 hpi and 48 hpi, while *SPs* showed a significant up-regulation at 48 hpi. In normal protective immune responses, SPs are responsible for activating the proteolytic cascade of immune pathways, including the prophenoloxidase-mediated melanization pathway and the upstream signaling of the Toll pathway. In contrast, serpins function as the primary negative regulators of this process to prevent excessive immune activation and tissue damage. This dysregulated expression pattern of these antagonistic genes suggests a disruption of host immune homeostasis during the progression of acute mycosis. The differential expression genes in *E. grisescens* larvae at 48 h after *M. anisopliae* infection were significantly enriched in drug metabolism, CYP450, and oxidative phosphorylation pathway, indicating that the anti-toxic genes were enriched to participate in detoxification and antioxidant effects to protect the body from damage of toxins produced by spores and new mycelia in the middle and late stages of infection [69,70,71]. Enrichment of up-regulated genes in the AMPK signaling pathway, reactive oxygen species metabolic process, and xenobiotic metabolism by CYP450 further reflects the activation of broad stress and immune-related responses in *E. grisescens* larvae upon *M. anisopliae* challenge.

Amino acids are important molecules for the executive functions of living organisms and can also be used as energy sources under stress conditions [72]. Metabolome analysis results showed that the metabolism of arginine, phenylalanine, proline, tryptophan, and tyrosine were down-regulated, while the cysteine, methionine, glycine, serine, and threonine metabolism pathways were up-regulated in the treatment group. Arginine was one of the main amino acids in the synthesis of bioactive polypeptides and played an important role in improving the body’s immunity, which could be converted into phosphoarginine by using arginine kinase (AK) [73,74]. Although the abundance concentration of arginine was decreased in response to *M. anisopliae*, the abundance of arginine derivatives, such as alanylarginine and histidinyl-Arginine was increased, which may be involved in the host’s immune regulation. Tyrosine was the phenoloxidase (PO) substrate initially present in insect plasma and it was rapidly metabolized by PO, which leads to melanin [75,76]. Both transcriptome and metabolome analysis results indicated that most DEGs and DAMs in *E. grisescens* larvae in responses to *M. anisopliae* were significantly enriched in tyrosine metabolism (ko00350), suggesting tyrosine metabolism played important roles in the response of tea geometrid to pathogenic fungi (Figure S2). Meanwhile, the decrease in intermediary metabolism substance in the tyrosine metabolism pathway was detected in the treatment group, indicating that cell death appeared in insects after infection with a lethal dose of *M. anisopliae* spore, which is consistent with the high mortality observed in the late stage of infection.

Insect AMPs were synthesized by the insect fat body and secreted into the hemolymph, regulated by the Toll signaling pathway and Imd signaling pathway, which were the main immune effector of insect innate immune defense system [77,78]. Transcriptome analysis results showed that multiple genes in the Toll-like receptor and Imd signaling pathway were up-regulated in the *M. anisopliae* infection group, indicating these immunomodulatory pathways were activated, which drives the transcriptional regulation of AMP genes. Consistent with this, we observed persistent up-regulation of attacin and gloverin genes at both 24 hpi and 48 hpi, as well as up-regulation of cecropin-like gene expression at 48 hpi. However, functional classification of these AMPs reveals distinct target specificities: attacins are reported to specifically target Gram-negative bacteria, while gloverins have confirmed activity against Gram-negative bacteria, Gram-positive bacteria, and viruses, with no direct antifungal activity described for either peptide in Lepidoptera to date [79,80,81]. The significant up-regulation of these two AMPs is more likely to reflect an antibacterial immune response against gut Gram-negative bacteria, rather than an antifungal response to *M. anisopliae*. Previous studies have demonstrated that entomopathogenic fungal infection can disrupt the host midgut microbiota homeostasis, leading to the overproliferation of Gram-negative bacteria, which in turn triggers the up-regulation of bacteria-targeting AMPs such as gloverin and attacin [82]. In contrast, cecropin-like peptides have been widely reported to possess direct antifungal activity in lepidopteran insects [83]. In our study, the cecropin-like gene showed no significant up-regulation at 24 hpi and only a slight increase at 48 hpi, indicating that the host did not initiate an effective antifungal effector response during the observed period of acute mycosis. This is consistent with previous findings that only the activation of fungus-specific effector molecules can be used as a reliable marker of functional antifungal immune response, rather than the up-regulation of broad-spectrum or bacteria-specific AMPs [84]. Meanwhile, the up-regulation of intermediate metabolites in the cysteine and glycine metabolism pathways in infected larvae may reflect the activation of amino acid metabolism to support the synthesis of various AMPs during the infection process.

## 5. Conclusions

In this study, physiological and biochemical changes in *E. grisescens* larvae infected by a highly virulent strain of *M. anisopliae* were analyzed based on transcriptome and metabolome analysis by high-throughput sequencing and LC-MS/MS to identify the regulated genes and metabolites. Comprehensively, complicated regulation processes were triggered in *E. grisescens* larvae after *M. anisopliae* infection, such as pattern recognition, innate immune signaling pathways, and detoxification systems. Specifically, we found that the host’s immune response during the acute mycosis stage (24–48 hpi) is dominated by an antibacterial response, characterized by significant up-regulation of the bacteria-specific AMPs attacin and gloverin. In contrast, the antifungal effector cecropin-like peptide showed only slight up-regulation at 48 hpi, indicating that the host failed to initiate an effective protective antifungal immune response during the observed infection period. The synchronous up-regulation of serine proteases and their inhibitory serpins further reflects the disruption of host immune homeostasis during the progression of lethal fungal infection. Meanwhile, the widespread activation of detoxification-related genes (including *GSTs*, *CYP450*, and antioxidant genes) and the dysregulation of amino acid metabolism were also prominent features of the host’s response to *M. anisopliae* infection. In addition, abnormal amino acid metabolism may lead to excessive energy consumption, impaired protein synthesis, cellular dysfunction, and cell death, ultimately contributing to host mortality. Overall, our findings delineate transcriptomic and metabolomic dynamics of *E. grisescens* in response to *M. anisopliae*-induced acute mycosis, clarify the functional specificity of different AMPs in the host response and provide new insights into the interaction between entomopathogenic fungi and lepidopteran hosts. These data also lay a foundation for understanding the response of non-model lepidopteran pests to fungal biopesticides and identifying potential targets for controlling the tea geometrid pest.

## Figures and Tables

**Table 1:** Summary of transcriptome sequencing data.

Sample	Clean Reads	Clean Base	GC (%)	Q20 (%)	Q30 (%)
CK_24h-1	29,313,279	8,779,377,306	48.83	98.01	94.41
CK_24h-2	25,220,515	7,553,664,762	47.28	97.87	94
CK_24h-3	25,832,542	7,737,358,694	47.21	98.15	94.61
Ma_24h-1	22,951,046	6,873,862,792	48.62	98.11	94.59
Ma_24h-2	22,311,315	6,681,626,028	47.44	98	94.3
Ma_24h-3	19,749,525	5,913,782,578	49.03	97.98	94.43
CK_48h-1	26,145,286	7,830,709,466	46.76	98.27	94.86
CK_48h-2	29,131,994	8,724,130,570	48.25	97.99	94.31
CK_48h-3	20,888,434	6,254,844,438	46.07	98.14	94.6
Ma_48h-1	21,058,663	6,305,053,186	50.47	97.8	94.13
Ma_48h-2	21,209,335	6,350,107,452	49.29	97.74	93.94
Ma_48h-3	20,687,278	6,194,087,194	47.51	97.99	94.33

**Table 2:** Changes of key genes in *E. grisescens* larvae after *M. anisopliae* infection for 24 h and 48 h.

Unigene ID	Gene Annotation	Expression Changes	
		Ma_24h	Ma_48h
Eg_11_047_0	*GNBP*	Up	Down
Eg_11_038_0	*GNBP*	Up	Down
Eg_15_0912_0	*βGRP*	Up	-
Eg_16_351_1	*PGRP*	Up	Up
Eg_01_642_1	*PGRP*	Up	Up
Eg_09_165_1	*PGRP*	Up	-
Eg_01_080_0	*SP*	-	UP
Eg_05_609_1	*SP*	Down	Down
Eg_31_015_1	*SP*	-	UP
Eg_12_433_0	*serpins*	-	UP
Eg_15_0578_1	*serpins*	Up	Up
Eg_19_534_1	*serpins*	Up	Up
Eg_28_073_1	*serpins*	-	UP
Eg_07_466_0	*serpins*	Up	Up
Eg_22_568_1	*spätzle*	-	UP
Eg_22_031_0	*spätzle*	Down	Up
Eg_24_195_1	*toll-like receptor*	-	UP
Eg_25_021_1	*toll-like receptor*	Down	Up
Eg_28_321_1	*toll-like receptor*	-	UP
Eg_28_328_1	*toll-like receptor*	Down	Up
Eg_19_018_0	*cactus*	-	UP
Eg_26_235_1	*pelle*	Up	-
Eg_25_138_0	*pellino*	Down	Down
Eg_25_139_0	*pellino*	-	DOWN
Eg_19_279_0	*janus kinase*	-	UP
Eg_17_469_1	*attacin*	Up	Up
Eg_28_283_1	*gloverin*	Up	Up
Eg_25_317_0	*PPO*	-	UP
Eg_01_711_1	*CY* *P450*	Up	Up
Eg_05_167_0	*CYP450*	Up	Up
Eg_05_168_0	*CYP450*	Up	Up
Eg_15_0450_0	*CYP450*	Up	Up
Eg_15_1042_0	*CYP450*	Up	Up
Eg_17_379_0	*CYP450*	Up	Up
Eg_21_006_1	*CYP450*	Up	Up
Eg_21_008_0	*CYP450*	Up	Up
Eg_21_331_0	*CYP450*	Up	Up
Eg_31_195_1	*CYP450*	Down	Up

**Table 3:** Changes of key metabolites in *E. grisescens* larvae after *M. anisopliae* infection for 24 h and 48 h.

Metabolite ID	Metabolite Name	KEGG Pathway Annotation	Ma_24h	Ma_48h
neg_8126	Quercetin	AMPK signaling pathway (ko04152)	Up	Up
neg_3449	Berberine		Up	-
pos_13597	L-Arginine	Arginine and proline metabolism (ko00330)	Down	Down
neg_9059	Alanylarginine		Up	Up
pos_10142	Histidinyl-Arginine		Up	Up
neg_7642	S-Adenosylhomocysteine	Chemical carcinogenesis-reactive oxygen species (ko05208)	Up	Down
neg_4019	Hydroquinone		Up	Down
neg_1943	Hydroxyhydroquinone		Up	-
neg_14600	Docosanedioic acid	Cutin, suberine, and wax biosynthesis (ko00073)	-	Up
neg_11968	16-Hydroxypalmitic acid		-	Up
pos_10406	5′-S-Methyl-5′-thioinosine	Cysteine and methionine metabolism (ko00270)	-	Up
neg_3172	N-Formylmethionine		-	Up
neg_6504	Lidocaine	Drug metabolism-cytochrome P450 (ko00982)	-	Up
neg_8298	6-Thioxanthine 5′-monophosphate	Drug metabolism—other enzymes (ko00983)	Up	Up
pos_195	6-Methylthioguanosine monophosphate		-	Up
neg_6116	Bis(glutathionyl)spermine	Glutathione metabolism (ko00480)	Up	Up
neg_5316	Creatine	Glycine, serine and threonine metabolism (ko00260)	Up	Down
pos_401	Betaine		-	Up
pos_895	D-Glycerate		-	Up
neg_4808	alpha-[3-[(Hydroxymethyl) nitrosoamino]propyl]-3-pyridinemethanol	Metabolism of xenobiotics by cytochrome P450 (ko00980)	Up	Up
pos_979	4-[(Hydroxymethyl)nitrosoamino]-1-(3-pyridinyl)-1-butanone		Up	Up
neg_8001	S-(Formylmethyl)glutathione		Up	Up
pos_13459	2-Phenylacetamide	Phenylalanine metabolism (ko00360)	Down	Up
neg_7098	trans-Cinnamate		Down	Down
neg_3189	L-Tryptophan	Tryptophan metabolism (ko00380)	Down	Down
neg_3186	Tryptamine		Down	Down
pos_2422	4-Maleylacetoacetate	Tyrosine metabolism (ko00350)	-	Down
pos_4051	L-Metanephrine		Down	Down
neg_5054	3-Methoxytyramine		Down	-
neg_3833	4-Coumarate	Ubiquinone and other terpenoid-quinone biosynthesis (ko00130)	Up	Down
neg_1330	4-Coumaroyl-CoA		-	Up
neg_6126	p-Coumaric acid		Up	-

## Data Availability

The original contributions presented in this study are included in the article/Supplementary Material. Further inquiries can be directed to the corresponding authors.

## Supplementary Materials

The following supporting information can be downloaded at: https://www.mdpi.com/article/10.3390/insects17030262/s1, Figure S1: Expression profiles of four AMP genes in *E*. *grisescens* larvae at 24 and 48 hpi following *M*. *anisopliae* treatment, Figure S2: Integrated transcriptome−metabolome KEGG pathway enrichment analysis in *E*. *grisescens* larvae 48 hpi after *M*. *anisopliae* treatment. The circle color indicates the number of DEGs; the circle size represents the number of DAMs. Table S1: Mortality statistics of *E. grisescens* larvae at 48 h post-infection with different concentrations of *M. anisopliae* conidia, Table S2: Probit analysis of mortality probability for *E. grisescens* larvae at 48 h post-infection with *M. anisopliae* conidia.
