# Supplementary figures and images for: Immune and Metabolic Responses in Ectropis grisescens Infected by Metarhizium anisopliae: Insights from Transcriptome and Metabolome Analyses
Source: Insects. 2026 Feb 28;17(3):262. doi: 10.3390/insects17030262 (PMC13027307; doi:10.3390/insects17030262)

Figure S1

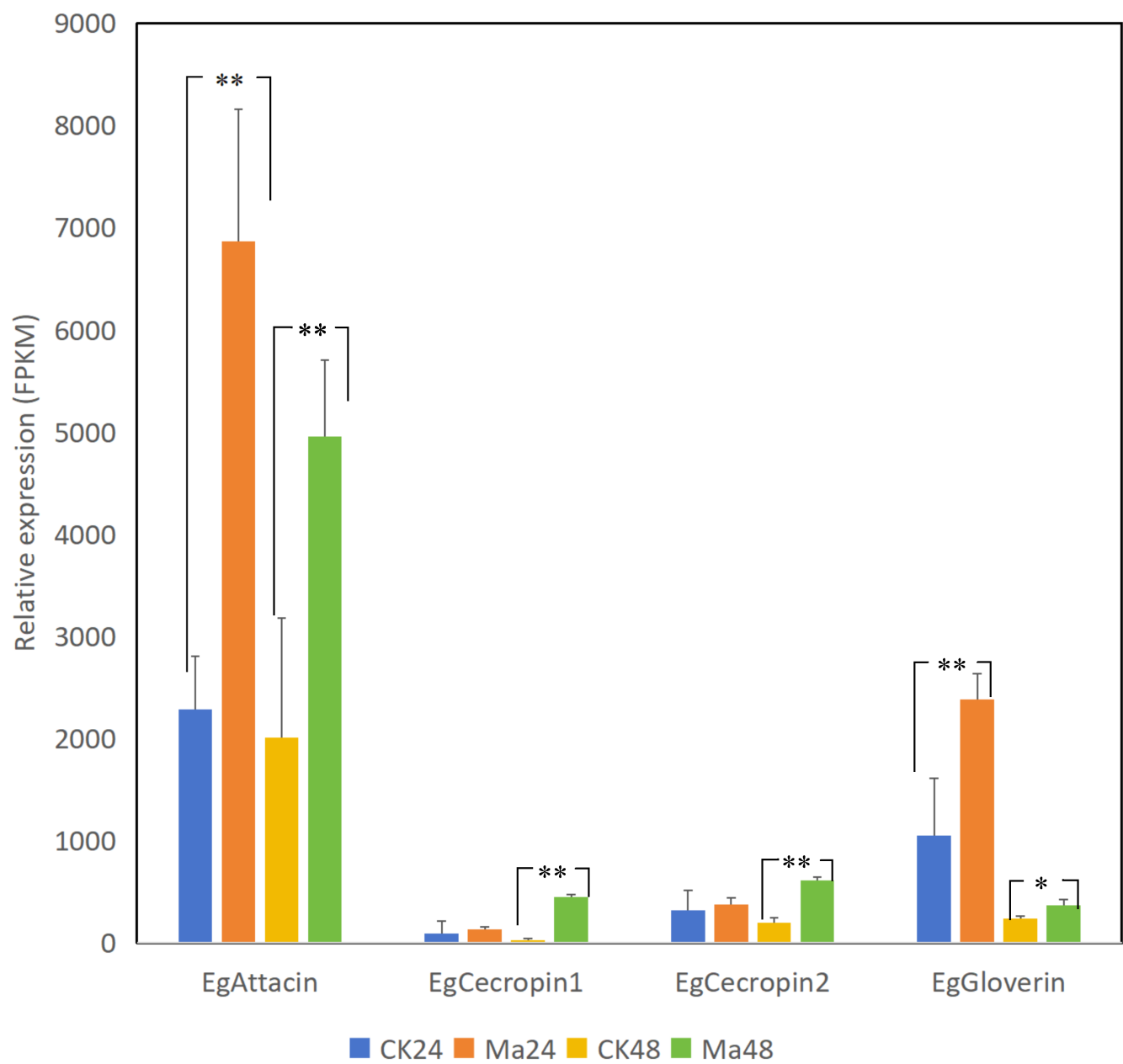

Figure S2

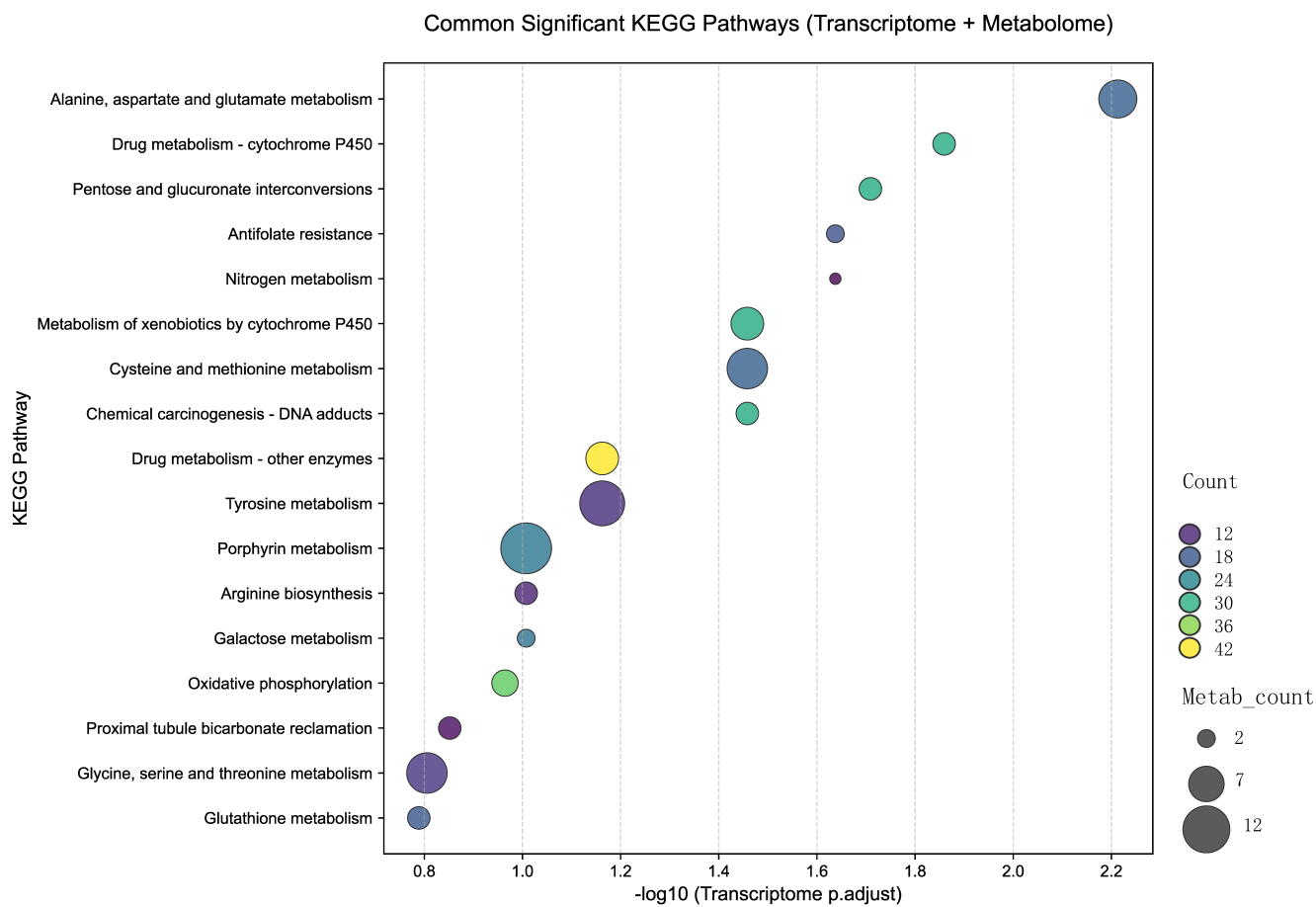

Supplement: Supplementary file 1 [file insects-17-00262-s001.zip › Supplementary Figs.pdf]
